# The Function of Selenium in Central Nervous System: Lessons from MsrB1 Knockout Mouse Models

**DOI:** 10.3390/molecules26051372

**Published:** 2021-03-04

**Authors:** Tengrui Shi, Jianxi Song, Guanying You, Yujie Yang, Qiong Liu, Nan Li

**Affiliations:** 1 Shenzhen Key Laboratory of Marine Biotechnology and Ecology, College of Life Sciences and Oceanography, Shenzhen University, Shenzhen 518055, China; 18792600863@163.com (T.S.); 1800251022@email.szu.edu.cn (J.S.); 2060251029@email.szu.edu.cn (G.Y.); yangyujie2016@email.szu.edu.cn (Y.Y.); liuqiong@szu.edu.cn (Q.L.); 2 The Central Laboratory, Shenzhen Second People′s Hospital, the First Affiliated Hospital of Shenzhen University Health Science Center, Shenzhen 518035, China; 3 Shenzhen-Hong Kong Institute of Brain Science, Shenzhen 518060, China; 4 Shenzhen Bay Laboratory, Shenzhen 518055, China

**Keywords:** selenium, MsrB1, central nervous system, redox, synaptic plasticity

## Abstract

MsrB1 used to be named selenoprotein R, for it was first identified as a selenocysteine containing protein by searching for the selenocysteine insert sequence (SECIS) in the human genome. Later, it was found that MsrB1 is homologous to PilB in *Neisseria gonorrhoeae*, which is a methionine sulfoxide reductase (Msr), specifically reducing L-methionine sulfoxide (L-Met-O) in proteins. In humans and mice, four members constitute the Msr family, which are MsrA, MsrB1, MsrB2, and MsrB3. MsrA can reduce free or protein-containing L-Met-O (*S*), whereas MsrBs can only function on the L-Met-O *(R)* epimer in proteins. Though there are isomerases existent that could transfer L-Met-O *(S)* to L-Met-O *(R)* and vice-versa, the loss of Msr individually results in different phenotypes in mice models. These observations indicate that the function of one Msr cannot be totally complemented by another. Among the mammalian Msrs, MsrB1 is the only selenocysteine-containing protein, and we recently found that loss of MsrB1 perturbs the synaptic plasticity in mice, along with the astrogliosis in their brains. In this review, we summarized the effects resulting from Msr deficiency and the bioactivity of selenium in the central nervous system, especially those that we learned from the MsrB1 knockout mouse model. We hope it will be helpful in better understanding how the trace element selenium participates in the reduction of L-Met-O and becomes involved in neurobiology.

## 1. Introduction

The oxidation of free L-methionine (L-Met) to L-methionine sulfoxide (L-Met-O) by chemical agents, such as iodine, iodate, and hydrogen peroxide, was first demonstrated in 1938 [1]. Because of the chirality of sulfur, two diastereomers, L-Met-O *(S)* and L-Met-O *(R)*, will be formed in equal volumes when L-Met is oxidized. Later, it was found by Bernett that the growth of rats is restrained when the L-Met in their diet is replaced by L-Met sulfone, whereas, it is not obstructed by L-Met-O [2]. Because free L-Met-O is unable to be inserted into polypeptides during protein synthesis, because methionyl-tRNA synthetase does not recognize it [3]. These observations indicate that L-Met-O but not sulfone could probably be converted back into L-Met in a mechanism, which was unknown at that point. 

In the 1970s, the L-Met-O residue in protein was detected, for example, in the human crystallin lens during development of senile nuclear cataracts [4]. Further study showed that the consequence of L-Met oxidation of many proteins inhibited their functions [5,6], and there is a thioredoxin- (Trx) and thioredoxin reductase (TXNRD)-dependent mechanism that could convert L-Met-O back to L-Met [7,8]. Finally, in 1981, scientists obtained an enzyme which could reduce L-Met-O when they were studying the Escherichia coli (*E. coli*) ribosome protein 12, a protein which loses its activity upon oxidation of selected L-Met residues by hydrogen peroxide [9]. This enzyme was named peptide L-Met-O reductase [10,11] (for review, please refer to [12]).

**Figure 1:**
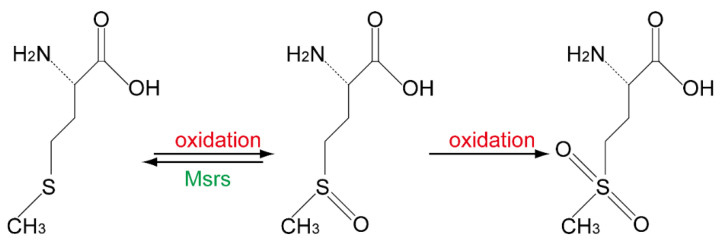
Methionine residue can be oxidized into methionine sulfoxide by, e.g., ROS and further oxidized into methionine sulfone by, e.g., performic acid; however, only methionine sulfoxide can be reduced back into methionine by methionine sulfoxide reductase (Msr) in a stereospecific manner.

With the development of genetic cloning technology, in the early 1990s, scientists successfully identified MsrA in bovines and humans that are homologous to the *E. coli* peptide L-Met-O reductase, and found that MsrA is able to reduce both free and protein-bound L-Met-O [13,14,15]. During the same time, an MsrA and Trx homologous protein in *Neisseria gonorrhoeae* called PilB was found to be enzymatically active toward both L-Met-O *(R)* and L-Met-O *(S)* [16]. This further lead to the identification of human MsrB1 and MsrB2. MsrB1 was discovered first as a selenoprotein [17,18]; its homology to PilB made scientists rename it from selenoprotein R/X to MsrB1. Meanwhile, MsrB2 was first named CBS-1, for it was identified by searching for the PilB similar genes by using the c-DNA library of the human ciliary body [19]. Thereafter, the stereoselectivity of Msrs was reported. MsrA was found to stereo-specifically reduce L-Met-O *(S)* [20], whereas, MsrB could only reduce L-Met-O *(R)* in proteins [21]. Following the discovery of MsrB3 [22], the mammalian Msr family has been carefully studied during the past two decades. Many Msr gene knockout (KO) mouse models have been developed. Each model displays unique features, indicating their functions could not be compensated by one another. Previously, we observed that the loss of MsrB1 impairs the spatial learning activity of mice, which is very similar to the phenotype derived from selenium-deficient diet feeding mice. In this review, we focused on summarizing the observations we obtained from MsrB1 KO mice, with the expectation of helping readers and ourselves to better understand how the trace element selenium becomes involved in regulating L-Met-O reduction (Figure 1) and synaptic plasticity. 

## 2. Loss of Msr Resulted in Different Phenotype in Mice

MsrA in mammals is widely distributed in the cytoplasm, nucleus, and mitochondria. Whereas MsrB1 is in cytoplasm and nucleus, MsrB2 is found in the matrix of mitochondria, and MsrB3 is mainly found in the endoplasmic reticulum (Table 1). Moreover, in isolated primary neurons, astrocytes, and microglia from mice brains, by using q-PCR to examine the mRNA levels of Msr, it was found that all Msrs are highly expressed in astrocytes, especially MsrB2 and MsrB3, and the mRNA levels of these two in astrocytes are 10 times higher than those in neurons and microglia. However, the mRNA level of MsrB1 in astrocytes is similar to in microglia and both of them are only slightly higher than that in neurons [23]. 

The knockout of MsrA in mice leads to difficulties in learning complex tasks, such as in operant learning tests, in which MsrA^−/−^ mice manifest significantly slower learning of how to press levers multiple times to receive rewards than wild-type mice. This is probably because of the degeneration of neurons in the hippocampal areas, and the abnormal dopamine levels in brain tissue, along with lower locomotive activities. These mice also exhibit a tip-toe walking pattern after six months of age [24]. In hyperbaric oxygen conditions, the cytochrome C in the lens of MsrA deficiency mice is readily oxidized at Met-65 and Met-80 residues, leading to the aggregation and decomposition of cytochrome C, and eventually the development of cataracts [25]. Moreover, MsrA knockout has been reported to shorten the lifespan of mice [26], but this observation is quite controversial [27]. Recently, it has also been shown that MsrA knockout mice exhibit progressive hearing loss and sensitivity to acoustic trauma [28].

In 2013, it was reported that MsrB1 could reduce oxidized actin, thus rescuing the polymerization activity of actin in vitro [29]. However, loss of MsrB1 in mice did not severely perturb the development, though the levels of malondialdehyde, protein carbonyl and methionine sulfoxide, lipid peroxidation, and oxidized glutathione were significantly increased in their livers and kidneys [30]. Moreover, the deficiency of MsrB1 exacerbates acetaminophen-induced hepatotoxicity represented by increased hydrogen peroxide production, lipid peroxidation, and protein oxidation levels [31]. Meanwhile, after being treated by LPS, the anti-inflammatory cytokines produced by macrophages are reduced in MsrB1 knockout models [32]. Recently, it was observed that the loss of MsrB1 induces astrogliosis in mouse brains, along with an impairment of spatial learning activity. The brain slices of these mice displayed downregulated long-term potentiation (LTP) due to the dephosphorylation of CaMKII α/β [23].

MsrB2 KO mice models have also been developed recently. Global KO in mice decreases platelets [33]. The depletion of MsrB2 platelets leads to reduced mitophagy and increased platelet apoptosis because of the oxidation of Parkin. In terms of MsrB3, it has been found that MsrB3 knockout induces static-ciliary tract degeneration and cochlear hair cell apoptosis, which eventually results in hearing loss in mice [34] (Table 1). These observations indicate that each Msrs has some unique functions and cannot be completely replaced by others. Though each of the three MsrBs can reduce L-Met-O *(R)*, the substrates of each are different.

**Table 1:** The phenotypes of Msrs knockout (KO) mice.

Msrs	Substrate	Subcellular Localization	Phenotype of KO Mice
MsrA	L-Met-O *(S)* in or free of proteins [35]	Cytoplasm, nucleus, mitochondria [36,37]	Learning disability, motor behavior disorders, progressive hearing loss [28]
MsrB1	L-Met-O *(R)* in proteins	Cytoplasm, nucleus [30]	Oxidative stress increase in kidney and liver [30], learning and memory disability [23]
MsrB2	L-Met-O *(R)* in proteins	Mitochondria [38]	Increased platelet apoptosis [33]
MsrB3	L-Met-O *(R)* in proteins	Endoplasmic reticulum [22]	Hearing loss [34]

## 3. Deficiency of Selenium or Selenoproteins Results in Disfunction of the Brain

Selenium used to be considered as a toxic chemical, for it was involved in the “alkali disease” in livestock [39] and excessive selenium intake caused hair and nail loss in humans [40]. However, this viewpoint was overturned upon the discovery of the essentiality of this element in rats [41] and *E. coli* [42]. Thereafter, it was found that selenium could be synthetized into proteins via Selenocysteine (Sec), the 21st amino acid in nature, which is encoded by the stop codon UGA [43]. 

In terms of Sec, it cannot be simply regarded as a cysteine in which the element sulfur is replaced by selenium. In fact, synthesis of Sec starts on the phosphorylated-serine-tRNA. HSe^-^ is catalyzed into SeH_2_PO_3_^−^ by selenophosphate synthetase 2 (SEPHS2), then Sec synthase (SecS) connects SeHPO_3_^−^ and phosphorylated-serine-tRNA to form Sec-tRNA (for review, please refer to [44]). As mentioned above, Sec is encoded by UGA. To insert Sec-tRNA into this stop codon in the mRNA of the corresponding selenoprotein, a special stem–loop structure called a Sec insertion sequence (SECIS) in the 3’-untranslated regions of the mRNA is essential. The help of trans-acting factors, such as Sec-specific elongation factor (EFsec) and SECIS binding protein (SBP2), is also necessary (for review, please refer to [44]). To date, 25 selenoprotein genes have been characterized by searching for the SECIS in human genome, and their translation products include glutathione peroxidases (GPX)1~4,6 and TXNRD1~3 and iodothyronine deiodinases (DIO)1~3, SEPHS2, SELENOF, SELENOH, SELENOI, SELENOK, SELENOM, SELENON, SELENOO, SELENOP, SELENOS, SELENOT, SELENOV, SELENOW, and MsrB1 [45]. However, the functions of many of them are still obscure to date. 

As mentioned above, the reducing activity of Msrs is dependent on the existence of Trx and TXNRDs, as well as the cofactor NADPH. All of the human TXNRDs are selenoproteins. TXNRD1 is located in cytosol, while TXNRD2 is distributed in mitochondria. Both of them are widely expressed in variant tissues and cell types; however, TNDRD3 is only found in testes [46]. Due to the knockout of TXNRD1 [47] or TXNRD 2 [48], causing early embryonic death, neuronal cell line—specifically TXNDRD 1 or TXNRD2—depletion is needed to further demonstrate their impact on the brain.

Selenoprotein P is one of the best studied selenoproteins so far. It contains 10 selenocysteins in humans and is believed to be responsible for selenium transportation, especially for the retention of selenium by the brain [49]. Deletion of the mouse selenoprotein P encoding gene *SELENOP* remarkedly decreases brain selenium content [50,51,52]. In addition, *SELENOP* knockout results in altered hippocampus synaptic function represented by disrupted spatial learning activity. Moreover, the ablation of ApoER2, the receptor of selenoprotein P that facilitates its uptake, also leads to abnormal neurological consequences, which is similar to the phenotypes derived from selenoprotein P deficiency [53]. These observations are also in line with the results that synaptic transmission is altered in wild-type mice that have been fed with a selenium-deficient diet [52,54]. 

Besides selenoprotein P, selenoprotein T deficiency also showed serious influence in mice. Global knockout *SELENOT* resulted in embryonic death. Conditional depletion of *SELENOT* in neuron lead to reduced volume of different brain structures, including hippocampus, cerebellum, and cerebral cortex, and triggered a hyperactive behavior [55]. In addition, mutation of SecS, which catalyzed the formation of sec-tRNA, produced progressive cerebro-cerebellar atrophy (PCCA), an autosomal recessive disorder resulting in severe brain abnormalities [56]. These studies indicated that selenium and selenoproteins play important roles in brain development and functions.

## 4. The Mystery Underlying the Impairment of Synaptic Plasticity in Selenium-Deficient Mice

The levels of selenium in the liver and kidney are sensitive to dietary selenium, but the level of selenium in the brain can remain normal under the condition of low selenium in the diet. This is because the transport of selenium to the brain is mainly dependent on selenoprotein P and its receptor [57], whereas the levels of selenium in periphery organisms are directly from ingestion. As mentioned before, depletion of *SELENOP* and its receptor *ApoER2* results in decreased spatial memory ability in mice, as well as defects in synaptic transmission and LTP [52,53]. At present, the molecular mechanisms underlying these observations remain unclear. Given that the level of MsrB1 is quite dependent on the selenium diet supply [30], the deficiency of MsrB1 may be involved in the neurological disfunctions elicited by the knockout of *SELENOP* and its receptor *ApoER2*, as well as a selenium-deficient diet. It can be imagined that *SELENOP* knockout not only reduces selenium levels in brain tissue, but may also affect the expression of many selenium proteins, including MsrB1.

**Figure 2:**
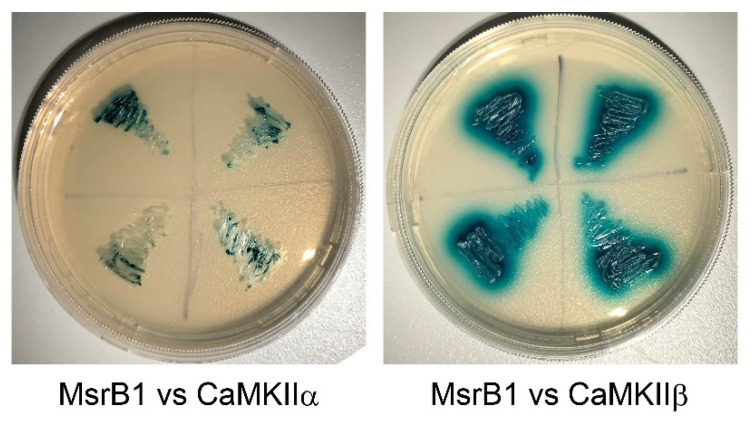
The interaction of MsrB1 with CaMKIIα and CaMKIIβ demonstrated by yeast two-hybrid screening.

Previously, MsrB1 was found to interact with clusterin (CLU) by yeast two-hybrid screening [58]. The expression of CLU is closely related to the occurrence of Alzheimer’s disease (AD) [59]. The results from another laboratory showed that MsrB1 can also interact with transient receptor potential channel M6 (TRPM6) [60]. When treated with hydrogen peroxide, Met1755 of TRPM6 is oxidized, while MsrB1 is able to protect the viability of TRPM6 and to reduce the damage caused by hydrogen peroxide [60]. Our previous research showed that in the hippocampus of *MsrB1* KO mice, the phosphorylation of CaMKIIα and CaMKIIβ was significantly decreased [23]. However, whether MsrB1 directly interacts with CaMKIIα and CaMKIIβ is unknown. Thus, recently, we further tested the interaction of murine MsrB1, in which selenocysteine was mutated to cysteine with CaMKIIα and CaMKIIβ by yeast two-hybrid screening (Figure 2). It was shown that MsrB1 could indeed interact with both CaMKIIα and CaMKIIβ directly.

It had been well established that synaptic plasticity, which could be represented by both LTP and long-term depression (LTD) in electrophysiology methods, is quite dependent on the phosphorylation of CaMKIIα and CaMKIIβ [61]. Ca^2+^ influx could induce the autophosphorylation of CaMKIIα and CaMKIIβ at Thr286/Thr286, respectively. In turn, the activation of CaMKII enhanced the synaptic activity of amino-3-hydroxy-5-methyl-4-isoxazoleprotionic acid receptors (AMPAR), thereby strengthening the LTP [62]. Most methionine oxidation is known to disrupt the normal function of proteins; however, the CaMKII is activated by methionine oxidation and it can be reduced by MsrA [63]. Though, the activity induced by oxidation is much lower than that is triggered by phosphorylation. 

**Figure 3:**
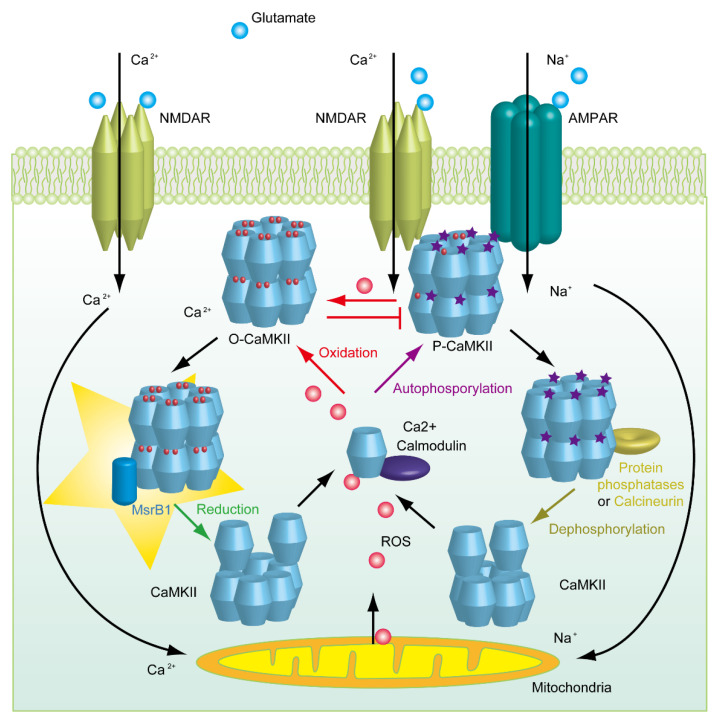
Schematic description of the hypothesis of how MsrB1 is involved in synaptic plasticity. The autophosphorylation of CaMKII could be triggered by Ca^2+^/calmodulin in an excited neuron. However, the overload of Ca^2+^ could induce excitatory toxicity, including the production of ROS by mitochondria, which could further oxidize CaMKII and perturb the phosphorylation of CaMKII. Therefore, synaptic plasticity would be impaired by excessive ROS. Meanwhile, MsrB1 could reduce the oxidized methionine residue in CaMKII and subsequently rescue the synaptic plasticity.

It is worth noting that the oxidation site of CaMKIIα and CaMKIIβ at Met280/281 is very close to their autophosphorylation site Thr286/287, respectively. It has also been detected that prolonged exposure to nitric oxide impairs CaMKII activity by reducing the autophosphorylation at Thr286 [64]. By collecting this evidence and our observations in MsrB1 KO mice, we propose that MsrB1 is involved in regulating synaptic plasticity by reducing oxidized CaMKIIα and CaMKIIβ. As shown in the schematic description (Figure 3), a transmitter such as glutamate activates the ion channel n-methyl-d-aspartate receptor (NMDAR) and induces Ca^2+^ influx. Ca^2+^/calmodulin further triggers the autophosphorylation of CaMKII, which could subsequently enhance the synaptic activity by recruiting AMPAR. However, excessive ions could induce the production of ROS by mitochondria. As a result, the overloaded ROS oxidizes CaMKII, which perturbs the autonomous modulation of CaMKII. In case of impairing the function of CaMKII, MsrA and MsrB1 in cytosol need to exert their reductive activity to restore the function of CaMKII. Therefore, in the following scenarios, such as the deficiency of selenium in the diet, the KO of *SELENOP* and its receptor, as well as the loss of MsrB1 or MsrA, synaptic plasticity is broken.

## 5. Perspectives

Due to the high expression level of MsrB1 in the liver and kidney, more attention has been paid to its protective effect under oxidative stress previously. However, recent studies have shown that the expression of MsrB1 in vitro can reduce the methionine sulfoxide at positions 44 and 47 residues of actin. Met44/47 of actin can be oxidized by Micals into L-Met-O, thereby causing changes in spatial conformation and inhibiting its aggregation ability [65], while MsrB1 can restore the aggregation ability of actin by reducing these L-Met-O [29]. However, it is not clear whether Micals and MsrB1 are involved in regulating neuronal plasticity through mediating the redox of actin.

Many clues indicate that Msrs plays a very important role in the central nervous system and is closely related to the occurrence and development of neurodegenerative diseases. For example, when MsrA is depleted in Alzheimer’s disease (AD) model mice, the level of amyloid-beta (Aβ) significantly increases, indicating that MsrA can directly regulate the oxidation state of Aβ and transform soluble Aβ into aggregated Aβ. It is generally believed that soluble Aβ oligomer has more severe neuronal toxicity, thus MsrA has neuronal protective activity during the pathological process of AD [66]. In addition, Adams et al. reported that MsrB3 is one of the genes related to hippocampus formation and volume. In the normal human hippocampus, MsrB3 is highly expressed in pyramidal neurons of stratum lucidum in the CA3 region, but less in the CA1 region, while the expression level in the neurons of the CA1 region is increased in AD patients and AD rat models [67].

AD is one of the most dramatic neurodegenerative diseases, and according to onset time, AD is divided into early-onset/familial AD and late-onset/sporadic AD. The former group accounts for approximately 5% of the total number of AD patients, those who develop AD at an early age (24–65 years) and often carry genetic mutations associated with excessive production of Aβ, especially Aβ 1–42, such as the mutation in Aβ precursor protein (APP) and APP shearing enzyme presenilin-1/2 [68]. The latter group usually develop AD after the age of 65, accounting for ~95% of all AD patients and making up the largest group of dementia patients. Through genome-wide association studies and other methods, it has been found that many genes related to lipid metabolism, immune response, and endocytosis are correlated with the occurrence of late-onset AD, including APOE, TREM2, PICALM, and CLU. The exact relationship between these proteins’ mutation with AD remains not fully understood, but some experiments have shown that most of these mutations cause Aβ clearance dysfunction [69]. Thus, the "amyloid cascade hypothesis" has long been dominant in the study of AD etiology. Taking multiple technologies, such as FRET, Co-IP, and pull down, it had been found that MsrB1 can directly interact with Aβ1–42, suggesting a high possibility that MsrB1 may affect the aggregation capacity of Aβ through the regulation of oxidative modification of Aβ [70], which is very similar to the function of MsrA.

The drugs that inhibit the production and aggregation of Aβ failed to achieve clinical success in curing AD [71,72]. On the way for looking for new therapeutic targets of AD, calcium hypothesis was introduced, proposing that the destruction of the calcium steady state is a major cause of AD, and it was reported that calcium concentration in endoplasmic reticulum is highly increased in AD patients [73]. This further leads to the dysfunction of the downstream signal path and LTP/LTD, finally resulting in the loss of synapses and the degeneration of neurons [74]. Neurons are very sensitive to calcium concentrations, and even a slight disorder of the calcium level would cause neurological dysfunction [75,76]. A variety of calcium channels exist on the membrane, such as voltage-gated Ca^2+^ channels (VGCCs), calcium releasing-activated channels (CRACs), and non-selective cation channels, such as NMDAR, AMPAR, transient receptor potential (TRP), ryanodine sensors (RyR), and 1,4,5-inositol trisphosphate receptor (IP3R). Among them, the effect of Aβ on NMDAR has been widely studied. It has been shown that NMDAR is overactivated in the early stage of AD, resulting in an increase of the calcium concentration in neurons [77]. Memantine, a non-competitive inhibitor of NMDAR has thus been approved for AD treatment by the Food and Drug Administration (FDA) [78]. 

Previous studies have shown that adding selenomethionine to AD mice diet can effectively reduce the deposition of Aβ in the brain, inhibit Tau phosphorylation by regulating GSK3β activity, and promote the removal of Tau through autophagy pathway [79,80,81], thus improving the cognitive and memory abilities of AD model mice. It is also noteworthy that the methylation of PP2A, which could mediate the dephosphorylation of Tau [82], at the L309 of its catalytic subunit can potentially increase its activity [83]. However, it was reported recently that sodium selenite decreases the methylation of PP2A [84]. In addition, the selenium supplement could effectively inhibit ROS-mediated apoptotic neural precursor cell death [85] and promote the neurosphere viability, development, and differentiation [86]. This suggests that selenoproteins may improve the pathological process of AD through multiple pathways. However, since the function of some selenoproteins in the central nervous system is still unclear, the related molecular biological mechanism needs to be further explored. Moreover, it has also been reported that the supplementation of selenium does not forestall dementia in clinical research [87], and combined with our unpublished proteomics data, we speculate that the disagreement of these observations may be because of the different forms of selenium that were used in these studies. Therefore, this demands attention on the differences between the ingestion of inorganic and organic selenium and their subsequent biological effects in this field. Moreover, AD mice models are artificially enforced to express mutated genes derived from familial AD, while ~95% of AD patients are late-onset sporadic cases, as mentioned before, and the etiopathology of sporadic cases may be distinct from that of familial cases. Thus, the effects of selenium supplementation for AD mice models and crowds of people may be different.

Many studies about Msrs indicate that the reduction and clearance of methionine sulfoxide is essential for maintaining the normal function of the central nervous system. Our previous results showed that MsrB1 is highly expressed in neurocytes and the deficiency of MsrB1 perturbs spatial learning and LTP/LTD in mice [23], but the mechanism of exploration is not sufficient. In vitro, it has been demonstrated that MsrB1 could specifically reduce methionine sulfoxide at positions 44 and 47 residues of actin, thus restoring its ability to polymerize into F-actin. Additionally, if MsrB1 could also exert such a direct role in vivo, then MsrB1 may play an important role in regulating neuronal synaptic formation, immune cell migration, tumor cell proliferation, and other pathological processes. Therefore, further exploration of the physiological role of MsrB1 will be helpful for understanding the function of selenium in the central nervous system and the treatment of neurodegenerative diseases.

## Data Availability

Please The data are available from the authors upon request.

## Sample Availability

Samples of the mutated MsrB1 plasmid is available from the authors.
